# Dedicator of cytokinesis 8 (DOCK8) mutation impairs the differentiation of helper T cells by regulating the glycolytic pathway of CD4^+^ T cells

**DOI:** 10.1002/mco2.747

**Published:** 2024-09-25

**Authors:** Panpan Jiang, Siyu Zhao, Xiaoyu Li, Shiyan Hu, Shuhan Chen, Yinming Liang, Lichen Zhang, Liaoxun Lu, Guofeng Fang, Lu Yang, Yanmei Huang, Heather Miller, Fei Guan, Jiahui Lei, Chaohong Liu

**Affiliations:** ^1^ Department of Pathogen Biology School of Basic Medicine Tongji Medical College and State Key Laboratory for Diagnosis and Treatment of Severe Zoonotic Infectious Diseases Huazhong University of Science and Technology Wuhan China; ^2^ Department Immunology School of Medicine Yangtze University Jingzhou China; ^3^ Center of Disease Model and Immunology Hunan Academy of Chinese Medicine Changsha China; ^4^ Laboratory of Genetic Regulators in the Immune System School of Medical Technology Xinxiang Medical University Xinxiang China; ^5^ Cytek Biosciences R&D Clinical Reagents Fremont California USA

**Keywords:** differentiation, DOCK8, glycolytic, T cell

## Abstract

Dedicator of cytokinesis 8 (DOCK8) deficiency is a primary immunodeficiency disease caused by mutations in exon 45 of the *DOCK8* gene. The clinical signs primarily consist of increased serum IgE levels, eczema, repeated skin infections, allergies, and upper respiratory tract infections. Using CRISPR/Cas9 technology, we generated a *DOCK8* exon 45 mutation in mice, mirroring the mutation found in patients. The results indicated that *DOCK8* mutation impairs peripheral T cell homeostasis, disrupts regulatory T cells (Tregs) development, increases ICOS expression in Tregs within peripheral lymph nodes (pLn), and promotes Th17 cell differentiation within the spleen and pLn. Upon virus infection, *DOCK8* mutation CD4^+^ T cells have a Th2 effector fate. RNA‐bulk sequencing data revealed alternations in the mTOR pathway of *DOCK8* mutant CD4^+^ T cells. We observed that *DOCK8* mutation upregulates the glycolysis levels in CD4^+^ T cells, which is related to the Akt/mTOR/S6/HIF‐1α pathway. In summary, our research elucidates that DOCK8 regulates the differentiation of helper T cells by modulating the glycolytic pathway in CD4^+^ T cells, thereby advancing the comprehension and offering potential treatment of diseases in DOCK8‐deficient patients.

## Graphical abstract

*DOCK8* mutation impairs the homeostasis of peripheral T cells, disrupts the development of Treg cells, increases ICOS expression in Tregs of the peripheral lymph nodes, promotes the differentiation of CD4^+^ T cells into Th17 cells, and upregulates the glycolysis levels in CD4^+^ T cells.

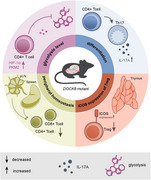

## 1 INTRODUCTION

Dedicator of cytokinesis 8 (DOCK8) is a protein belonging to the DOCK family, encoded by the *DOCK8* gene. It functions as a guanine nucleotide exchange factor (GEF), regulating the activity of the GTP enzymes of the Rho family.^1^ DOCK8 is crucial in the regulation of the actin cytoskeleton.^2^ It is predominantly expressed in immune cells and hematopoietic stem cells. DOCK8 is also expressed in nonimmune tissues including the kidney, lung, and pancreas.^3^ DOCK8 deficiency broadly affects the development, migration, growth, and adhesion of immune cells,^4^, ^5^, ^6^ thus impacting innate and adaptive immune responses.^7^, ^8^, ^9^, ^10^, ^11^ On the human level, *DOCK8* mutations give rise to autosomal recessive (AR) Hyper‐IgE Syndrome (AR‐HIES) accompanied by combined immunodeficiency (CID), which was first reported in 2004.^12^ This finding identified DOCK8 immunodeficiency syndrome (DIDS) as a clinical entity.^13^ Mutations in *DOCK8* lead to severe allergic reactions, refractory viral skin infections in AR‐HIES, and leukemia.^14^, ^15^, ^16^ Furthermore, there is no specific treatment for DIDS, and the main treatments are early diagnosis and immunological assessments.^17^

Numerous studies have shown that DOCK8 deficiency affects the survival, differentiation, and function of various T cell subsets. For instance, DOCK8 deficient mice and patients have a strong Th2 bias.^18^, ^19^ Th2 cells, which produce IL‐4, IL‐5, and IL‐13, are essential in combating extracellular parasites and facilitating allergic responses.^20^ DOCK8 deficiency also seriously affects Th17 and Th1 cell differentiation.^21^ Th1 cells, characterized by their production of IFN‐γ, are key to defending intracellular pathogens​. Th17 cells, known for secreting IL‐17 and IL‐22, are critical in protecting the body from extracellular bacteria and fungi while contributing to autoimmune diseases. *DOCK8* mutant mice have reduced numbers and shorter lifespans in naive CD8 T cells. After antigen stimulation, synaptic polarization was poorly displayed and cell division was delayed.^8^

Previous research has shown that helper T cell development and differentiation are influenced by metabolism,^22^ which acts as the regulator of T cell function and survival. Different T cell subsets need different energy and biosynthetic pathways to maintain unique functional requirements. For example, effector T cells are prone to using aerobic glycolysis, while naive T cells and Tregs rely on oxidative phosphorylation to supply energy needs.^23^ Upon antigen stimulation, naive T cells rapidly initiate metabolic pathways, upregulated glycolysis, and mitochondrial metabolism to enter the synthetic metabolic phase.^24^ Th17 cells exhibit high glycolytic activity and depend on pathways activated through mTOR and HIF‐1α. Additionally, Th17 cell development is hindered by HIF‐1α depletion or glycolysis inhibition.^25^ Therefore, understanding the metabolism of T cells might reveal insight into T cell subset differentiation in *DOCK8* mutant mice.

To develop *DOCK8* mutant mice that mimic the pathological state of *DOCK8* patients, CRISPR/cas9 technology was used to construct a *DOCK8* point mutation in exon 45. This *DOCK8* mutation destabilized the homeostasis of peripheral T cells, impeded the growth and ICOS expression of Treg cells within pLn, and promoted Th17 cell differentiation. RNA‐bulk sequencing data indicated that cell metabolism was crucial in the impact on CD4^+^ T cells with *DOCK8* mutation. Western blotting and seahorse experiments found that *DOCK8* mutation upregulated the level of glycolysis in CD4^+^ T cells, which was associated with the Akt/mTOR/S6/HIF‐1α pathway. In summary, our study elucidates that DOCK8 modulates helper T cell differentiation by altering glycolytic pathways in CD4⁺ T cells, providing insights into the molecular mechanisms underlying DOCK8 deficiency.

## 2 RESULTS

### 2.1 *DOCK8* mutation increases serum IgE levels and decreases DOCK8 protein in T cells

**FIGURE 1:**
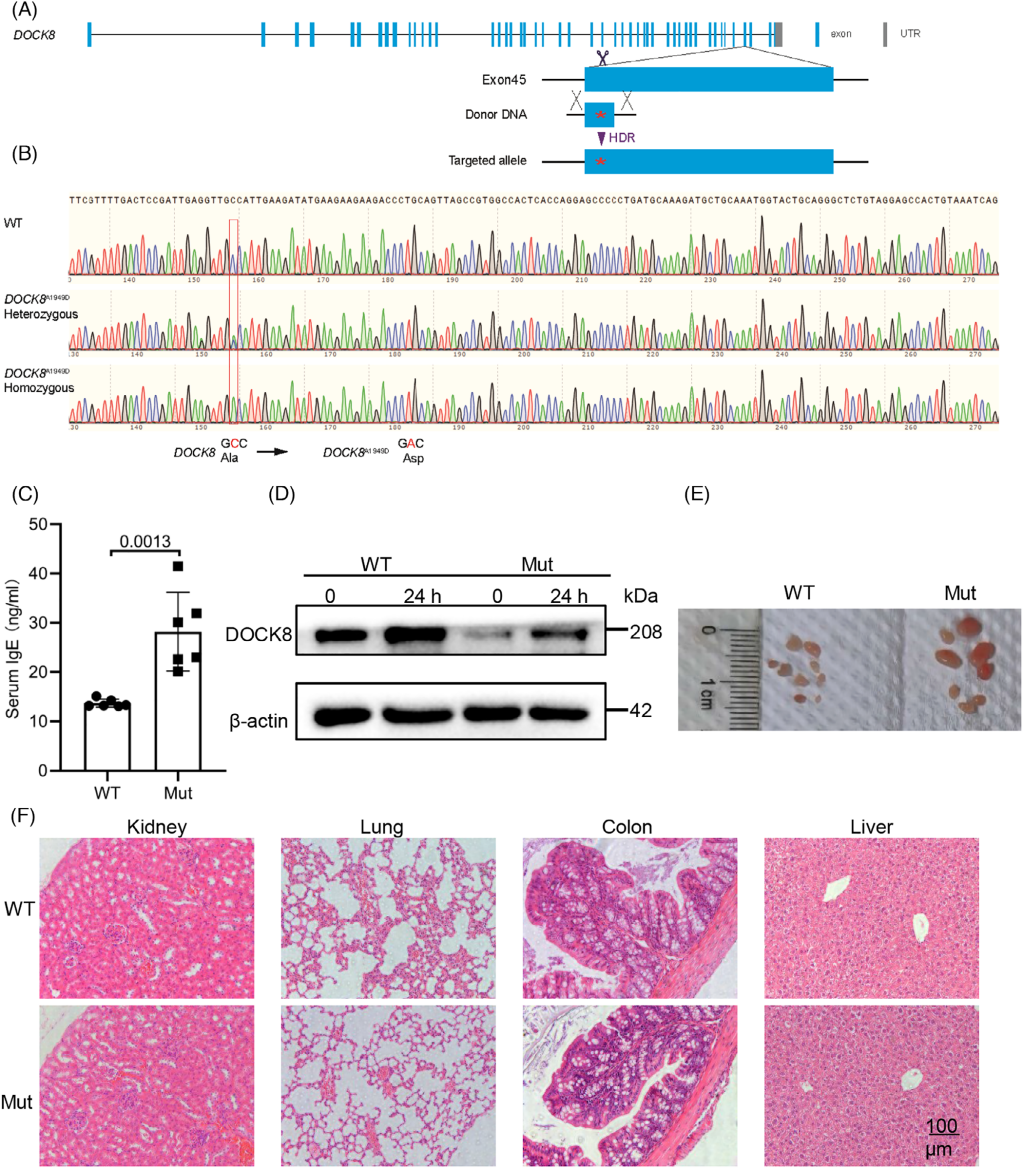
*DOCK8* mutation increases serum IgE levels and decreases dedicator of cytokinesis 8 (DOCK8) protein in T cells. (A,B) The DNA of *DOCK8* in wild type (WT) and *DOCK8* mutant mice was amplified by PCR and sanger sequencing (Mut = *DOCK8* mutant mice). (C) The level of IgE in serum was analyzed by enzyme linked immunosorbent assay (ELISA) (*n* = 6). (D) Western blotting of DOCK8 in CD4^+^ T cells. (E) The size of the peripheral lymph nodes (pLn) was shown (*n* = 9). (F) H and E staining results of tissues (scale bar = 100 µm).

To substitute the alanine residue (encoded by GCC) with an aspartic acid residue (encoded by GAC) at position 1949 of the *DOCK8* gene (Transcript: ENSMUST00000025831.8 *DOCK8*‐201), we developed a repair oligonucleotide in conjunction with two single guide RNAs (sgRNAs) specifically targeting exon 45 of the *DOCK8* gene. A silent mutation was incorporated at amino acid position 1947 to prevent re‐cleavage by Cas9 following homologous recombination. To confirm that the sequence of *DOCK8* was changed in exon 45, PCR and sanger sequencing were employed. Sequence alignments were conducted using the basic local alignment search tool (BLAST) search on the NCBI website (Figure 1A,B). To verify that our constructed *DOCK8* mutation mouse model could mimic AR‐HIES, we examined serum IgE levels by enzyme linked immunosorbent assay (ELISA), which revealed that *DOCK8* mutant mice had increased levels of serum IgE (Figure 1C), suggesting that the patient's high IgE clinical phenotype was reproduced in this mouse model. AR loss‐of‐function mutations in the *DOCK8* cause DIDS and the mutations commonly lead to a lack of DOCK8 protein expression.^13^ We found that the *DOCK8* mutant mice had a significant decrease in DOCK8 protein expression in CD4^+^ and CD8^+^ T cells (Figure 1D and S1A,B). Therefore, the *DOCK8* mutation mouse model was successfully constructed. Finally, although no significant lymphocytic infiltration was observed in *DOCK8* mutant mice, we did find that they had larger pLn (Figure 1E,F), which is consistent with observations in DOCK8 Treg‐specific deletion mice.^26^ In summary, the *DOCK8* mutation increases serum IgE levels and decreases DOCK8 protein in T cells.

### 2.2 *DOCK8* mutation impairs peripheral T cell homeostasis without affecting thymus T cell development

**FIGURE 2:**
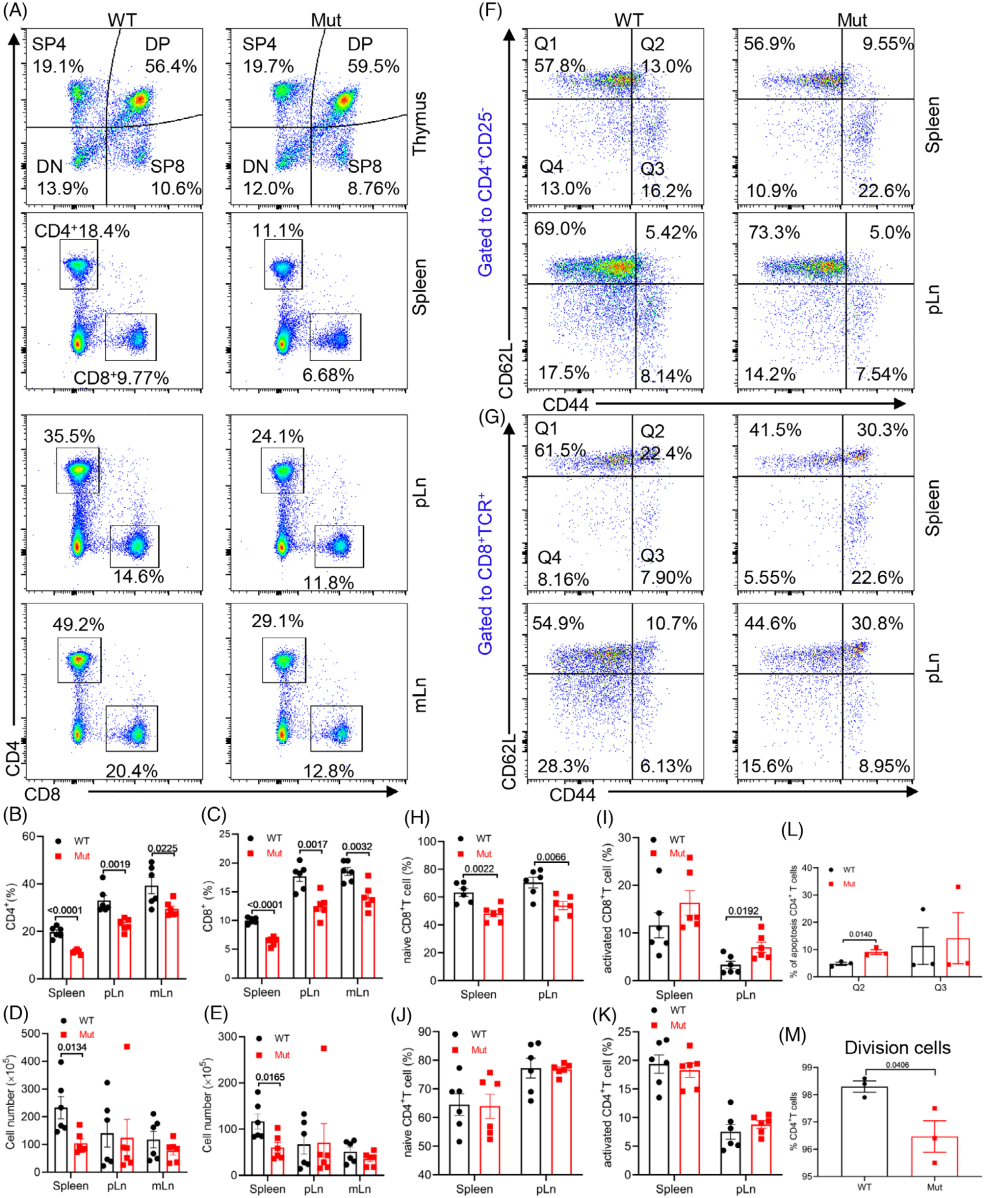
*DOCK8* mutation cripples peripheral T cell homeostasis without affecting thymus T cell development. (A–E) Flow cytometry analysis of T cells in the thymus, spleen, peripheral lymph nodes (pLn), and mesenteric lymph nodes (mLn). Representative plots of flow cytometry (A). Quantification of the percentages (B,C) and cell numbers (D,E) of CD4^+^ T cells and CD8^+^ T cells (SP4 ,  CD4 single positive; SP8, CD8 single positive; DP, double positive; DN, double negative; *n* = 6). (F–K) Flow cytometry analysis of naive and activated T cells in the spleen and pLn from WT and *DOCK8* mutant mice. Representative plots of flow cytometry (F,G). Quantification of the percentages of naive and activated CD8^+^ T cells (*n* = 6) (H,I). Quantification of the percentages of naive and activated CD4^+^ T cells (*n* = 6) (J,K). (L) Flow cytometry analysis of apoptotic CD4^+^ T cells in the spleen. Quantification of the percentages of late apoptotic cells (Q2) and early apoptotic cells (Q3, *n* = 3). (M) Proliferation of CD4^+^ T cells in the spleen after stimulation. Quantification of the division index by Flowjo software (*n* = 3).

Reports indicate that DOCK8‐deficient patients have reduced numbers of peripheral blood CD4^+^ and CD8^+^ T cells.^14^ To further validate the T cell immunophenotype of *DOCK8* mutant mice, we analyzed T cell expression in the thymus, spleen, pLn, and mesenteric lymph nodes (mLn) of *DOCK8* mutant mice. The results indicated that *DOCK8* mutant mice had significantly lower proportions of CD4^+^ and CD8^+^ T cells in the spleen, pLn, and mLn, along with a depletion of splenic CD4^+^ and CD8^+^ T cells, but the pLn and mLn CD4^+^ and CD8^+^ T cell numbers showed no significant difference (Figure 2A–E and Figure S2A). In contrast, no difference was found in the proportion and number of thymus in *DOCK8* mutant mice (Figure 2A and Figure S2B,C), suggesting that *DOCK8* mutation did not affect the development of thymic T cell.

Subsequently, we examined T cell activation in the *DOCK8* mutant mice. The findings revealed that, in *DOCK8* mutant mice, the proportion of naive CD44^low^CD62L^high^ CD8^+^ T cells in the spleen and pLn was distinctly lower, whereas notably higher in the thymus (Figure 2F–H and Figure S2D–I). However, activated CD44^high^CD62L^low^ CD8^+^ T cells in the pLn were significantly increased, whereas no significant difference was seen in the spleen, thymus, and mLn (Figure 2I and S2D–I). Moreover, no significant differences were detected in the proportion of naive and activated CD4^+^ T cells in the spleen and pLn (Figure 2J,K). Additionally, we noted an increase in late apoptotic CD4^+^ T cells in the spleen of *DOCK8* mutant mice (Figure 2L and Figure S2J). Finally, we examined the proliferation of CD4^+^ T cells. The results demonstrated reduced proliferation of CD4^+^ T cells in the spleen of *DOCK8* mutant mice (Figure 2M and Figure S2K). These results demonstrate that more peripheral T lymphocytes in *DOCK8* mutant mice are in an activated state and affect peripheral T cell homeostasis.

To test whether the reduced ratio of CD4^+^ and CD8^+^ T cells in *DOCK8* mutant mice was not influenced by the microenvironment of the mice and was intrinsic to the T cells, we constructed a mouse bone marrow chimeric model. To do this, the donor (CD45.2) bone marrow cells were mixed with the bone marrow cells of the CD45.1 recipient mice in a ratio of 1:1. The mixed cells were injected via the tail vein into the irradiated CD45.1 recipient mice (Figure S3A). Furthermore, the proportion of CD4^+^ and CD8^+^ T cells in the spleen and pLn of CD45.2^+^ *DOCK8* mutation donor mice was markedly reduced compared to CD45.2^+^ wild type (WT) donor mice (Figure S3B–H), which indicates that the *DOCK8* mutation affects the proportion of peripheral T cells intrinsically within T cells, rather than due to abnormal thymic T cell development.

### 2.3 *DOCK8* mutation disturbs the development and ICOS expression of Tregs in the pLn

**FIGURE 3:**
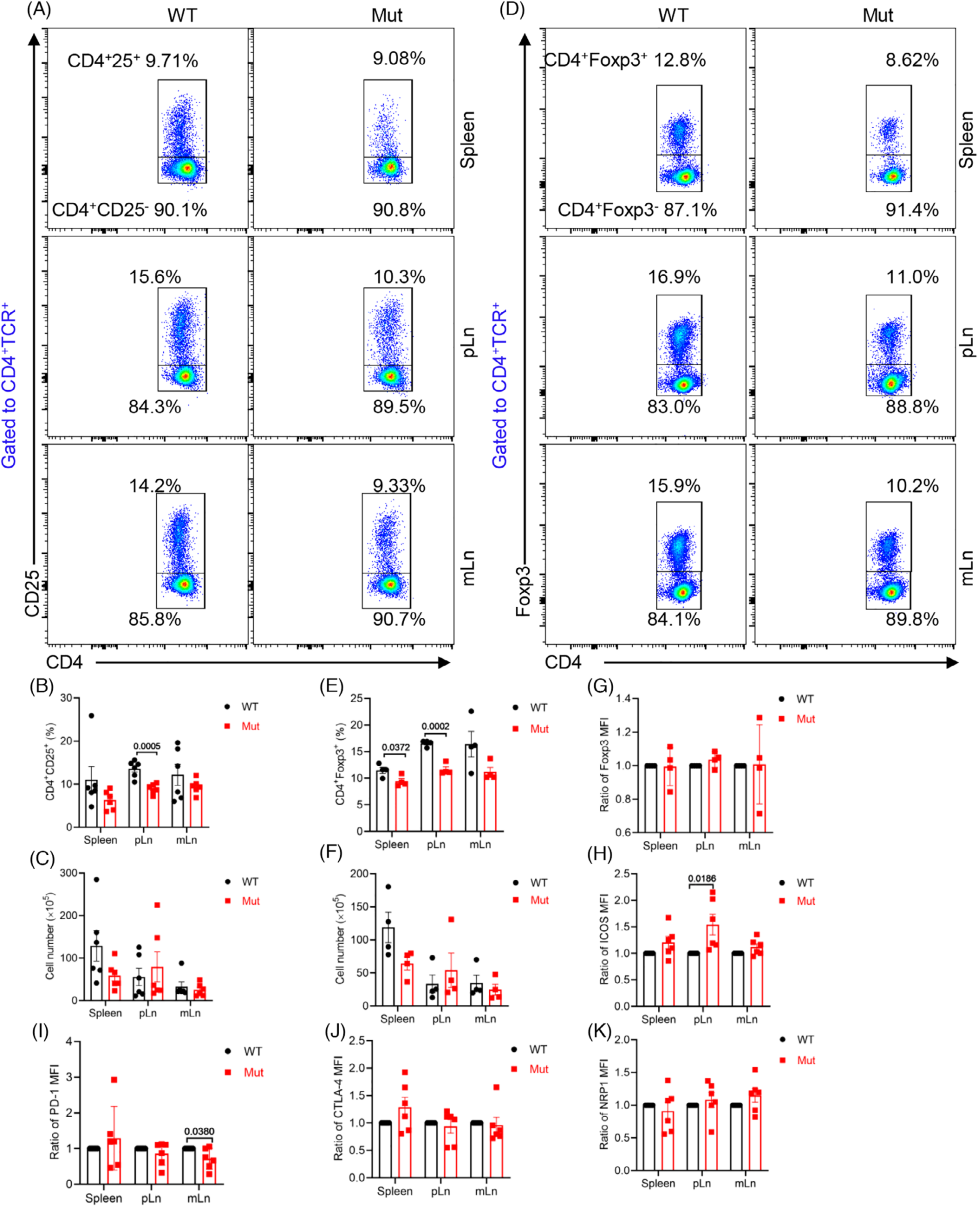
*DOCK8* mutation disturbs the development and ICOS expression of Treg cells in the peripheral lymph nodes (pLn). (A–K) Flow cytometry analysis of CD4^+^CD25^+^ Treg cells and CD4^+^Foxp3^+^ Treg cells in the spleen, pLn, and mesenteric lymph nodes (mLn). Representative plots of flow cytometry (A,D). The percentages and cell numbers of CD4^+^CD25^+^ Treg cells (B,C) and CD4^+^Foxp3^+^ Treg cells (E,F) were quantified (*n* = 6). The MFI of Foxp3 (G) was quantified in CD4^+^Foxp3^+^ Treg cells (*n* = 6). The MFI of ICOS (H), PD‐1 (I), CTLA‐4 (J), and NRP1 (K) was quantified in CD4^+^CD25^+^ Treg cells (*n* = 6).

Tregs are an essential subpopulation of T cells that mediate immune tolerance and regulate immune system homeostasis by maintaining peripheral T cell homeostasis, which inhibits the activation of auto‐reactive T cells. Studies have reported that Tregs are reduced and exhibited impaired suppressive activity in DOCK8‐deficient patients.^27^ Therefore, we investigated the effects of *DOCK8* mutation on Tregs subpopulations in the *DOCK8* mutant mice. The findings revealed that *DOCK8* mutant mice showed a significantly lower proportion of CD4^+^CD25^+^ and CD4^+^Foxp3^+^ Tregs in the pLn and a reduced proportion of CD4^+^Foxp3^+^ Tregs in the spleen. However, the number of CD4^+^CD25^+^ and CD4^+^Foxp3^+^ Tregs was not significantly changed, which might be due to the enlargement of the pLn (Figure 3A–F). These studies indicate a possible link between elevated activated T cells and a reduction in Tregs in *DOCK8* mutant.

To further investigate the mechanism of Tregs reduction, we first examined Foxp3, a key transcription factor specifically expressed by Tregs, which determines the development and function of Tregs as well as maintains homeostasis and suppresses the immune response effectively.^28^ The results showed that in the CD4^+^Foxp3^+^ Tregs of *DOCK8* mutant mice, there was no distinct difference in the mean fluorescence intensity (MFI) of Foxp3 (Figure 3G), suggesting that the reduction in Tregs was not due to a functional defect in Foxp3. Next, we detected the inhibitory molecules CTLA‐4, ICOS, PD‐1, and NRP1 that are expressed on the surface of Tregs and enhance their inhibitory function. The results showed that *DOCK8* mutant mice exhibited increased MFI of ICOS in pLn CD4^+^CD25^+^ Tregs, decreased MFI of PD‐1 in mLn CD4^+^CD25^+^ Tregs, and no significant difference in CTLA‐4 and NRP1 in CD4^+^CD25^+^ Tregs (Figure 3H–K). It has been shown that Tregs high expression of ICOS facilitates the maintenance of immune homeostasis.^29^ These results suggest that *DOCK8* mutations disrupt Tregs development and ICOS expression in the pLn, thereby affecting immune system homeostasis.

### 2.4 *DOCK8* mutation promotes Th17 cell differentiation

**FIGURE 4:**
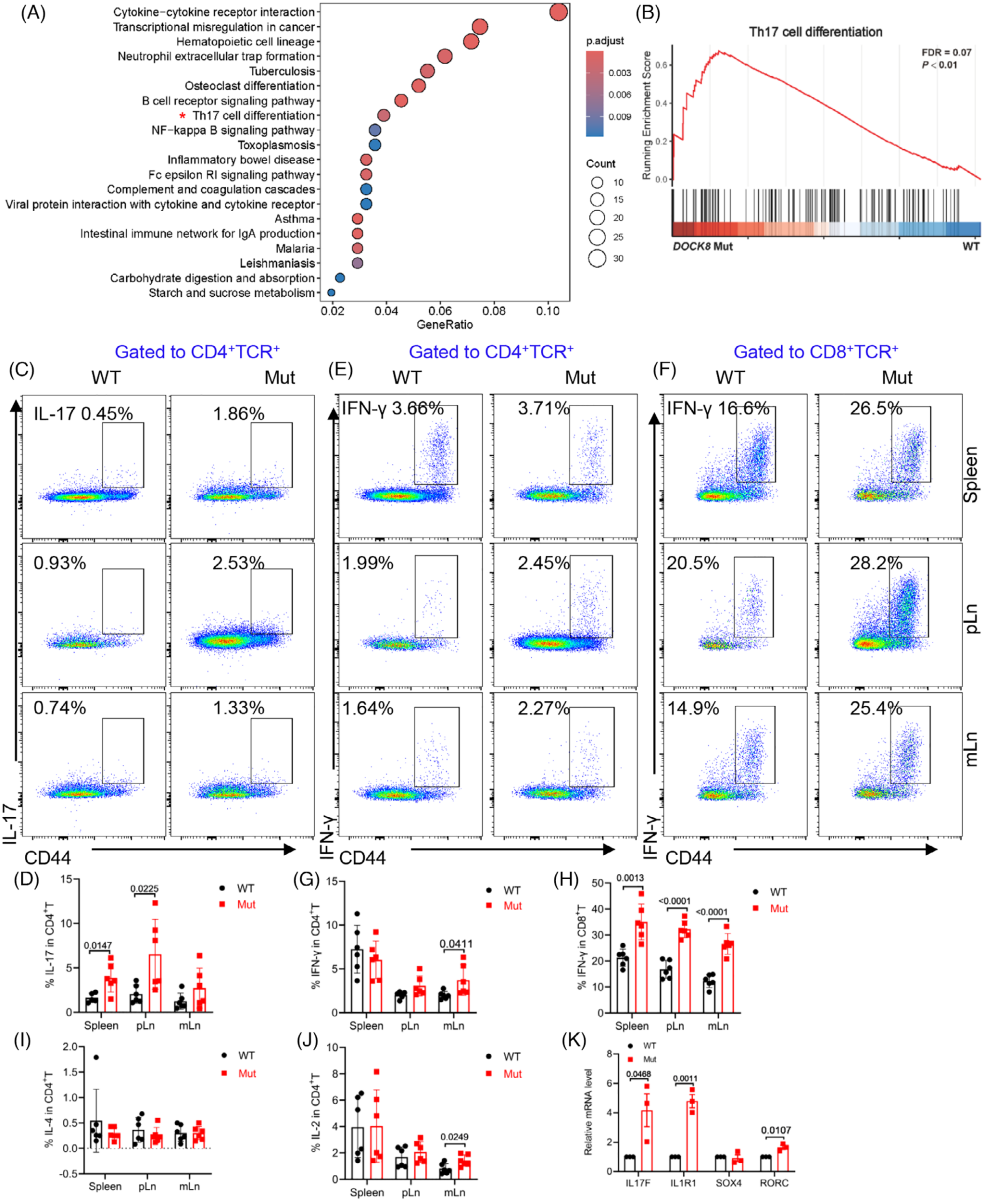
*DOCK8* mutation promotes CD4^+^ T cells differentiation to Th17 cells. (A) Kyoto encyclopedia of genes and genomes (KEGG) pathway enrichment analysis of remarkably different genes in CD4^+^ T cells (*p* < 0.05). (B) Gene enrichment analysis (GSEA) showing enrichment of the Th17 cell differentiation pathway in CD4^+^ T cells (*p* < 0.05). (C–J) Flow cytometry analysis of cytokine expression of CD4^+^ and CD8^+^ T cells in the spleen, peripheral lymph nodes (pLn), and mesenteric lymph nodes (mLn). Representative plots of flow cytometry (C,E,F). The percentages of IL‐17 (D), IFN‐γ (G,H), IL‐4 (I), and IL‐2 (J) were quantified in CD4^+^ and CD8^+^ T cells (*n* = 6). (K) The mRNA of *IL‐17F*, *IL1R1*, *SOX4*, and *RORC* was examined by RT‐PCR (*n* = 3).

To investigate the transcriptome changes in *DOCK8* mutation, we isolated CD4^+^ T cells for RNA‐bulk sequencing. We found that Th17 cell differentiation was significantly enriched in *DOCK8* mutant mice (Figure 4A). Also, gene enrichment analysis (GSEA) showed that genes on Th17 cell differentiation were significantly enhanced in CD4^+^ T cells from *DOCK8* mutant mice (Figure 4B), suggesting that *DOCK8* mutation might promote Th17 cell differentiation. To validate the hypothesis, we detected the levels of IFN‐γ, IL‐4, and IL‐17, which demonstrated that the proportion of IL‐17 by CD4^+^ T cells was increased in the spleen and pLn of *DOCK8* mutant mice (Figure 4C,D). The results were consistent with those of RNA‐bulk sequencing data. Elevated IFN‐γ by CD4^+^ T cells in mLn and by CD8^+^ T cells was also observed in *DOCK8* mutant mice (Figure 4E–H), whereas no significant difference was detected in IL‐4 and IL‐2 by CD4^+^ T cells in the spleen and pLn (Figure 4I,J). Furthermore, we also analyzed the RNA‐bulk sequencing data for IL‐17 producing related genes such as *RORC*, *SOX4*, *IL1R1*, and *IL‐17F*. We detected a significant elevated mRNA level of *RORC*, *IL1R1*, and *IL‐17F* in *DOCK8* mutant mice by RT‐PCR (Figure 4K). These results suggest that *DOCK8* mutation promotes Th1 and Th17 cell differentiation.

### 2.5 *DOCK8* mutation CD4^+^ T cells have a Th2 effector fate after lymphocytic choriomeningitis virus (LCMV) infection

**FIGURE 5:**
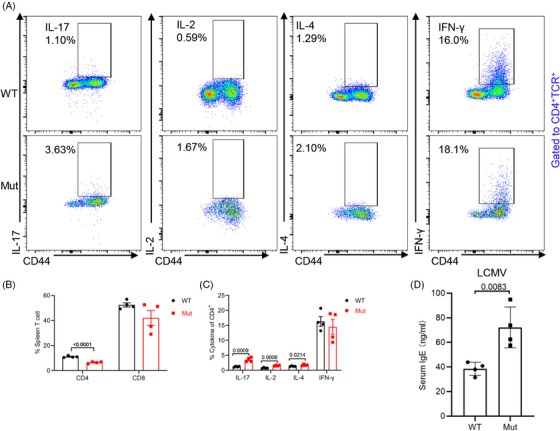
*DOCK8* mutation CD4^+^ T cells are biased to Th2 effector fate after lymphocytic choriomeningitis virus (LCMV) infection. (A–C) WT and *DOCK8* mutant mice were injected intraperitoneally with LCMV. One week later, flow cytometry analysis of cytokine expression was done in the spleen. Representative plots of flow cytometry (A). The percentages of CD4^+^ and CD8^+^ T cells were examined in the spleen (*n* = 4) (B). The percentages of IL‐2, IL‐4, IL‐17, and IFN‐γ were analyzed in CD4^+^ T cells (*n* = 4) (C). (D) The level of IgE in serum was quantified by ELISA after LCMV infection (*n* = 4).

Viral infections are frequently observed in DOCK8‐deficient patients.^14^ To find out the role of *DOCK8* mutation in viral pathogenesis, the level of cytokines in CD4^+^ T was examined in the spleens of WT or *DOCK8* mutant mice following LCMV infection. The findings revealed that *DOCK8* mutant mice had markedly lower proportions of CD4^+^ T cells after LCMV infection (Figure 5A,B). Unsurprisingly, IL‐2, IL‐4, and IL‐17 secreted by *DOCK8* mutation CD4^+^ T cells were increased after LCMV infection but IFN‐γ levels remained unchanged (Figure 5C). Meanwhile, ELISA experiments demonstrated that the expression of IgE in serum also increased after LCMV infection (Figure 5D). Together, our findings indicate that *DOCK8* mutation CD4^+^ T cells are biased toward Th2 effector fate after LCMV infection.

### 2.6 *DOCK8* mutation upregulates the glycolysis level of CD4^+^ T cells associated with Akt/mTOR/S6/HIF‐1α pathway

**FIGURE 6:**
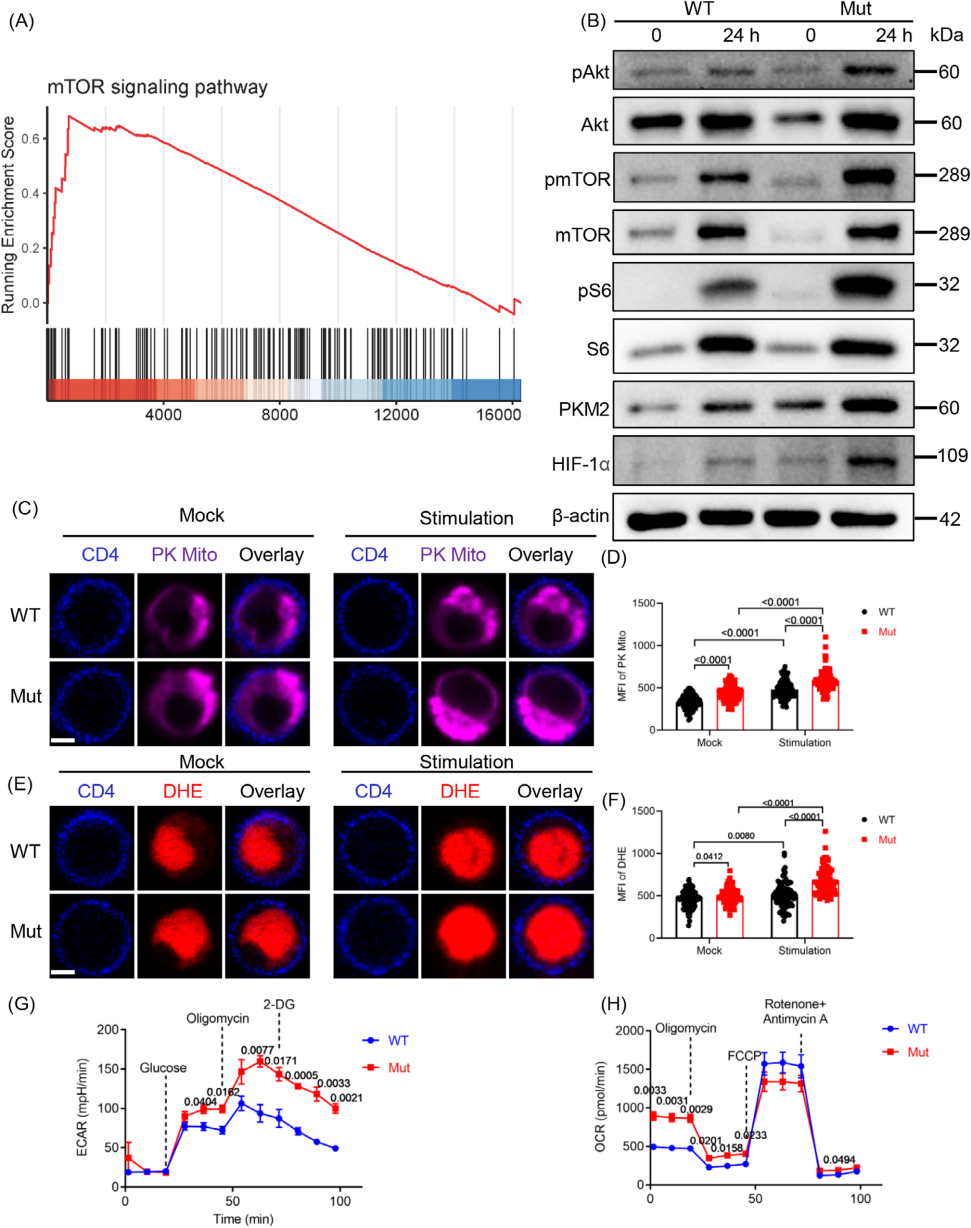
*DOCK8* mutation upregulates the glycolysis level of CD4^+^ T cells associated with Akt/mTOR/S6/HIF‐1α pathway. (A) Gene enrichment analysis (GSEA) showing enrichment of the mTOR signaling pathway in CD4^+^ T cells (*p* < 0.05). (B) Western blotting of pAkt, Akt, pmTOR, mTOR, pS6, S6, PKM2, and HIF‐1α in CD4^+^ T cells. (C–F) The mean fluorescence intensity (MFI) of PK Mito (C,D) and dihydroethidium (DHE) (E,F) were analyzed by confocal, respectively. (G) Extracellular acidification rate (ECAR) detection of CD4^+^ T cells (*n* = 3). (H) Oxygen consumption rate (OCR) detection of CD4^+^ T cells (*n* = 3).

Th1 and Th17 cell differentiation is regulated by mTOR signaling.^30^ Transcriptomic data showed that GSEA‐enriched genes to the mTOR signaling pathway were enriched in CD4^+^ T cells from *DOCK8* mutant mice (Figure 6A). Furthermore, we detected the expression of mTOR pathway related molecules. The findings showed that protein levels of pAkt, pmTOR, and pS6 were also markedly elevated in CD4^+^ T cells of *DOCK8* mutant mice after TCR activation (Figure 6B), indicating that the *DOCK8* mutation upregulated mTOR signaling. Transcriptomic data suggest that starch and sucrose metabolism may be crucial in *DOCK8* mutant mice (Figure 4A). mTOR can upregulate HIF‐1α to promote glycolysis and induce Th17 cell differentiation.^31^ Furthermore, the protein expression of HIF‐1α and pyruvate kinase M2 (PKM2, an important enzyme in the glycolysis process) was significantly elevated in *DOCK8* mutation CD4^+^ T cells (Figure 6B). The above results indicate that *DOCK8* mutation affects CD4^+^ T cell metabolism.

To examine the role of *DOCK8* mutation in CD4^+^ T cell metabolism, the mitochondria morphology and reactive oxygen species (ROS) were observed by staining the PK Mito and dihydroethidium (DHE), respectively. *DOCK8* mutation CD4^+^ T cells showed remarkably increased MFI of PK Mito and DHE in both anti‐CD3 plus anti‐CD28 stimulated and unstimulated (Figure 6C–F). Additionally, extracellular acidification rate (ECAR) revealed that the basal glycolytic capacity of *DOCK8* mutation mice in CD4^+^ T cells was elevated by the addition of glucose, the maximal glycolytic capacity of the cells was elevated by the addition of oligomycin, and the glycolytic reserve and non‐glycolytic acidification were elevated by the addition of 2‐Deoxy‐D‐glucose (2‐DG) (Figure 6G). This suggests that DOCK8 inhibits the CD4^+^ T cell glycolytic pathway. The results of oxygen consumption rate (OCR) showed that basal respiration of *DOCK8* mutation CD4^+^ T cells was significantly elevated, the oxygen consumption rate of oxidative phosphorylation was elevated by the addition of oligomycin, and maximum respiration was not significantly different by the addition of FCCP, but spare respiratory capacity and non‐mitochondrial oxygen consumption were elevated after the addition of rotenone and antimycin A, indicating that DOCK8 inhibits the oxidative phosphorylation of CD4^+^ T cells (Figure 6H). In conclusion, these results imply that *DOCK8* mutation upregulates the glycolysis level of CD4^+^ T cells associated with the Akt/mTOR/S6/HIF‐1α pathway.

## 3 DISCUSSION

In this study, we constructed a mouse model with the same exon 45 mutation as *DOCK8* patients using CRISPR/Cas9 technology to investigate the impact of *DOCK8* mutation on T cell differentiation. The proportions of CD4^+^ and CD8^+^ T cells in the spleen, pLn, and mLn of *DOCK8* mutant mice were markedly decreased, while T cells in the thymus were not affected. DOCK8‐deficient patients and mice had significantly reduced frequencies of T cells.^32^ Also, the spleen and pLn of *DOCK8* mutant mice exhibited decreased naive (CD44^low^CD62L^high^) and increased activated (CD44^high^CD62L^low^) T cell proportions, indicating that *DOCK8* mutation disrupts murine peripheral T cells homeostasis.

We discovered that the *DOCK8* mutation mice led to a decreased scale of both central and peripheral Treg cells, which was related to the increased expression of the inhibitory molecule ICOS. Studies report that *DOCK8* maintains the inhibition function of Treg cells by regulating the IL‐2 signal and can also enhance immune tolerance by forming immune synapses.^26^ Treg cells in DOCK8‐deficient patients show impaired suppressive activity.^27^ This inconsistency may be due to the maintenance of IL‐2 signaling. Therefore, whether DOCK8 regulates the function of Treg cells requires further investigation.

DOCK8‐deficient patients demonstrate hyper IgE and a lower proportion of CD4^+^ T cells, which secretes type‐2 cytokines. Similarly, DOCK8‐deficient mice have a type 2 differentiation bias in CD4^+^ T cells and elevated Th17 cells in the periphery.^18^, ^19^, ^32^, ^33^ Our study pointed out that CD4^+^ T cells in *DOCK8* mutant mice expressed more IFN‐γ and IL‐17, which are produced by Th1 and Th17, respectively, and participate in the antiviral immune response.^34^, ^35^ They also serve as significant pro‐inflammatory cytokines that facilitate the release of inflammatory mediators and are pivotal in various inflammatory and autoimmune conditions.^36^ After LCMV infection, *DOCK8* mutant mice had remarkably lower proportions of CD4^+^ T cells in the spleen, higher proportions of IL‐17, IL‐2 and IL‐4 produced by *DOCK8* mutation CD4^+^ T cells and elevated IgE levels in serum, indicating a shift of *DOCK8* mutation CD4^+^ T cells toward Th2 effector fate post‐LCMV infection.

Th17 and Treg cells generally maintain a dynamic balance of the immune system. Our study shows that the increase in Th17 cells and the decrease in Tregs further illustrate the impact of *DOCK8* mutation on immune homeostasis in mice. Also, Th17 cell differentiation was found to be very prominent by KEGG pathway enrichment analysis, further indicating that *DOCK8* mutation affects Th17 cell differentiation. The imbalance of the Treg/Th17 ratio is also a major pathogenic factor in infections and autoimmune diseases. The significant differential gene enrichment in CD4^+^ T cells in pathways such as asthma and inflammatory bowel disease is consistent with some clinical symptoms of DOCK8‐deficient patients, suggesting that the *DOCK8* mutation is related to these diseases. Therefore, *DOCK8* mutation may increase susceptibility to autoimmune disease in mice through upregulation of Th17 differentiation pathway.

Immunometabolism changes in cells have been shown to regulate the differentiation of CD4^+^ T cells into specific functional helper T cell subsets.^22^ Th1 and Th17 cell differentiation is mainly regulated by the mTOR pathway and the activation level of its downstream signaling molecule S6 is increased in the CD4^+^ T cells of *DOCK8* mutant mice. ^29^ In our study, the mTOR signaling pathway in CD4^+^ T cells of *DOCK8* mutant mice was significantly increased. Since mTOR can upregulate glycolytic metabolism pathway via HIF‐1α and PKM2,^30^ and Th17 cells have high glycolytic activity, we examined the metabolic pathway of *DOCK8* mutation T cells. We detected elevated levels of HIF‐1α and PKM2 in CD4^+^ T cells of *DOCK8* mutant mice. Also, the increase in ECAR further indicates that the glycolysis level of CD4^+^ T cells in *DOCK8* mutant mice is increased. Because the overexpression of HIF‐1α can inhibit mitochondrial respiration, we also tested this by detecting the OCR of cells. The oxidative phosphorylation level of CD4^+^ T cells in *DOCK8* mutant mice was increased. Therefore, DOCK8 regulates the mTOR/S6/HIF‐1α signaling to differentiate CD4^+^ T cells into subsets.

The current study had some limitations. Studies have indicated the critical role of DOCK8 in the survival and function of mouse and human CD8^+^ T cells.^8^ In our study, the DOCK8 protein expression in CD8^+^ T cells of *DOCK8* mutant mice was significantly reduced. However, the effect of *DOCK8* mutation on CD8^+^ T cell metabolism has not been reported yet. In a future study, we will explore the role of *DOCK8* mutation on glucose metabolism in CD8^+^ T cells. Additionally, no metabolic abnormalities have been reported in DIDS, while metabolic inhibitors have been reported widely.^37^, ^38^ Currently, small molecule inhibitors against metabolic targets are widely studied in anti‐tumor, cancer and anti‐viral.^39^, ^40^, ^41^, ^42^ The next aim of our in‐depth research project is to perform more metabolism‐related experiments on clinical samples to find metabolic inhibitors to treat DIDS.

In summary, the *DOCK8* mutation mouse model is suitable for studying DOCK8 deficiency, as it can replicate some of the patient's clinical manifestations and thus simulate the environment of the disease for potential molecular mechanism research. We also found that DOCK8 can affect the proliferation ability of CD4^+^ T cells and regulate their glucose metabolism. Taken together, these data show that DOCK8 can regulate the levels of glycolysis and oxidative phosphorylation by stabilizing the mTOR/HIF‐1α signaling pathway, thereby inhibiting the abnormal differentiation of Th17 cell and autoimmune disease development.

## 4 METHODS AND MATERIALS

### 4.1 Mice

All mice were fed under specific pathogen‐free (SPF) conditions with a 12 h light/dark cycle. All experiments were approved by the Animal Experiment Ethics Committee of Tongji Medical College (approval number, 3954).

### 4.2 Generation of DOCK8 mutation

To convert the GCC codon in exon 45 of the *DOCK8* gene (Transcript: ENSMUST00000025831.8 *DOCK8*‐201) from coding for an alanine residue to coding for aspartic acid at position 1949 of *DOCK8*, we designed a repair oligonucleotide along with two sgRNAs that target exon 45 of the *DOCK8* gene. The repair oligonucleotide sequence, with differences from the wild‐type C57BL/6 sequence indicated in lowercase and the protospacer sequences of the sgRNAs underlined, is as follows: TGGCCTCACTCTGCGGTTGTCTTCTGTTCAGTTCGTTTTGACTCCGATTGAaGTTGaCATTGAAGATATGAAGAAGAAGACCCTGCAGTTAGCCGTGGCCACTCACCA. The repair oligonucleotide was synthesized by BiOligo Biotechnology Co., Ltd., and the sgRNAs were provided by GenScript. Cas9 mRNA, sgRNAs, and the repair oligonucleotide were injected into mouse embryos as described previously.^43^ The genotyping of *DOCK8* ^A1949D^ knock‐in mice was performed by sequencing a 346‐bp DNA fragment flanking exon 45 using the following pair of PCR primers: forward 5′‐CCATCTCTGTGTGACCTGTTCA‐3′ and reverse 5′‐AACAAACAAGCCCTAACCAAGC‐3′.

### 4.3 Cells preparation

Mouse spleen, thymus, pLn, and mLn were collected and grounded. Cells were suspended in an HBSS solution containing 2% serum and then filtered with a nylon membrane after lysed red blood cells with Red Cell Lysis Buffer to obtain mononuclear cells. Reagents details are presented in Table S1.

### 4.4 Purification and activation of CD4+ T cell

Purified CD4^+^ T cells were isolated by murine CD4^+^ T cell Isolation Kit. For the activation of CD4^+^ T cells, sorted cells were cultured in 10 µg/mL plate‐bound anti‐CD3 plus anti‐CD28 for 5 h at 37°C as described previously.^44^ Reagents details are presented in Table S1.

### 4.5 Flow cytometry

For cell surface staining, mononuclear cells (1 × 10^6^) from the thymus, spleen, pLn, and mLn were stained with antibodies on ice for 30 min after incubation with the Fc blocker. For cell intracellular staining, cells (2 × 10^6^) were fixed, permeabilized, and subsequently cultured with antibodies on ice for 30 min. For cytokine analysis, mononuclear cells (2 × 10^6^) were cultured with PMA (50 ng/mL), GolgiStop (1:1000), and lonomycin (1 µM) at 37°C for 5 h. Then cells were collected and stained with antibodies on ice for 30 min. Finally, cells were measured using Attune NxT (Thermo Fisher, AFC2) and analyzed by FlowJo 10 software (TreeStar). Antibodies’ details are presented in Table S1.

### 4.6 Confocal

Purified CD4^+^ T cells (1.5 × 10^6^) were cultured with 10 µg/mL plate‐bound anti‐CD3 plus anti‐CD28 for 5 h at 37°C. To prepare ROS and PK Mito analysis, the cells were cultured with anti‐CD4 and DHE or PK Mito for 15 min at 37°C. Then, the cells were analyzed by a confocal microscope (Nikon). The MFI was analyzed using NIS‐elements AR 5.01. Over 50 individual cells were analyzed for confocal analysis. Reagents details are presented in Table S1.

### 4.7 Western blotting

Sorted and stimulated CD4^+^ T cells (2 × 10^6^) were lysed using RIPA buffer containing cocktail, NaF (1 M), and Na3VO3 (100 Mm). Then, cell lysates were examined by SDS‐PAGE, and immunoblotting was performed using antibodies (Bio‐Rad). Antibodies details are presented in Table S1.

### 4.8 Seahorse analysis

Purified CD4^+^ T cells were prestimulated in 24‐well plates with 10 µg/mL anti‐CD3 plus anti‐CD28 for 24 h at 37°C, and then seeded the cells into culture plates at 37°C for 1 h. For ECAR analysis, cells were stimulated with 2 uM Oligomycin, 10 mM Glucose, and 5 mM 2‐DG. For OCR analysis, cells were stimulated with 1 µM FCCP, 1.5 µM oligomycin, 1 µM antimycin A, and 500 nM rotenone as described previously.^45^ Finally, cells were examined by the Seahorse XFe24 Cell Metabolism Analyser (Agilent). Reagents details are presented in Table S1.

### 4.9 ELISA

Levels of IgE in serum were measured using a mouse IgE ELISA Kit. Reagents details are presented in Table S1.

### 4.10 LCMV infection

Mice were injected intraperitoneally with PBS containing 5 × 10^5^ PFU of LCMV‐Armstrong 53b (200 µL). One week later, the mice were executed, and T cells from the spleen were examined. The level of IgE in serum was tested by ELISA (INFINITE 200 PRO, TECAN).

### 4.11 BM chimeras

BM cells were collected from WT (CD45.1 recipients), *DOCK8* mutation (CD45.2, donor), and WT (CD45.2, donor) mice as described previously.^45^ After 8 weeks postinjection, spleen, thymus, and pLn cells were collected and isolated for staining by flow cytometry.

### 4.12 Transcriptome sequencing

For RNA‐seq libraries, VAHTS Universal V8 RNA‐seq Library Prep Kit for Illumina was constructed and then sequenced on the BGISEQ platform. Then, low‐quality reads were removed by the BGISEQ platform and aligned reads to the mouse reference genome.

### 4.13 RT‐PCR

In brief, total RNA was isolated from CD4^+^ cells (1 × 10^6^) using the trizol reagent and detected using real‐time reverse transcription PCR assay as described previously.^46^ The primer sequences refer to Table S2.

### 4.14 Statistics analysis

Data were presented as mean ± SEM and statistical analyses were presented using Prism 8.0 (GraphPad). When comparing two groups, an unpaired two‐tailed Student's *t*‐test was used. Asterisks indicate significant difference: *p < 0.05, ** *p* < 0.01, *** *p* < 0.001, **** *p* < 0.0001.

## AUTHOR CONTRIBUTIONS

Panpan Jiang and Siyu Zhao wrote the paper and performed the experiments and the statistical analysis. Xiaoyu Li, Shiyan Hu, Shuhan Chen, Lichen Zhang, Liaoxun Lu, and Yanmei Huang performed the flow cytometry, confocal experiments, and western blotting. Guofeng Fang performed RNA‐seq analysis. Yinming Liang, Lu Yang, Heather Miller, Fei Guan, and Jiahui Lei revised the manuscript. Chaohong Liu designed the research. All authors have read and approved the final manuscript.

## CONFLICT OF INTEREST STATEMENT

Heather Miller is an employee of Cytek Biosciences but has no potential relevant financial or non‐financial interests to disclose. The other authors declare no conflicts of interest.

## ETHICS STATEMENT

All animal experiments were approved by the Animal Experiment Ethics Committee of Tongji Medical College (approval number, 3954).

## Supporting information



Supporting Information

## Data Availability

The raw data of RNA‐bulk sequencing (No. CNP0006002) have been deposited in China National GeneBank DataBase (CNGBdb).
