# Supplementary material for: Dedicator of cytokinesis 8 (DOCK8) mutation impairs the differentiation of helper T cells by regulating the glycolytic pathway of CD4+ T cells
Source: MedComm (2020). 2024 Sep 25;5(10):e747. doi: 10.1002/mco2.747 (PMC11424684; doi:10.1002/mco2.747)
Supplement: Supplementary file 1 — Supporting Information [file MCO2-5-e747-s001.pdf]

Supplementary Materials for

Dedicator of cytokinesis 8 (DOCK8) mutation impairs the differentiation of helper T cells by regulating the glycolytic pathway of CD4+ T cells

Panpan Jiang *et al.*

*Corresponding author. Email: chaohongliu80@126.com

This PDF file includes:

Figures. S1 to S3

Tables S1 to S2

Figure. S1.

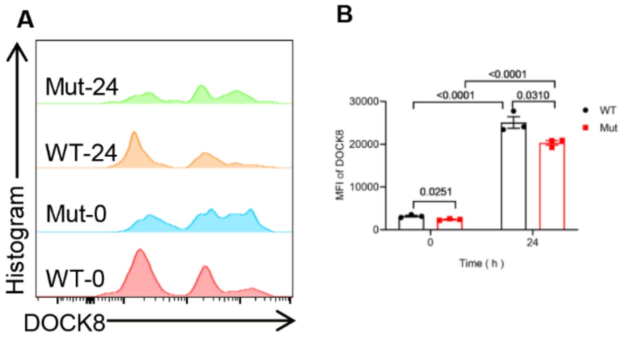

Figure S1. The level of DOCK8 protein in CD8⁺ T cells.

(A-B) CD8⁺ T cells were cultured in 10μg/ml plate-bound anti-CD3 plus anti-CD28 for 5 h at 37°C and the level of DOCK8 protein in CD8⁺ T cells of WT and *DOCK8* mutant mice was detected by flow cytometry. The data shown are the mean value ± SEM. **P* < 0.05; ***P* < 0.01; ****P* < 0.001.

Figure. S2.

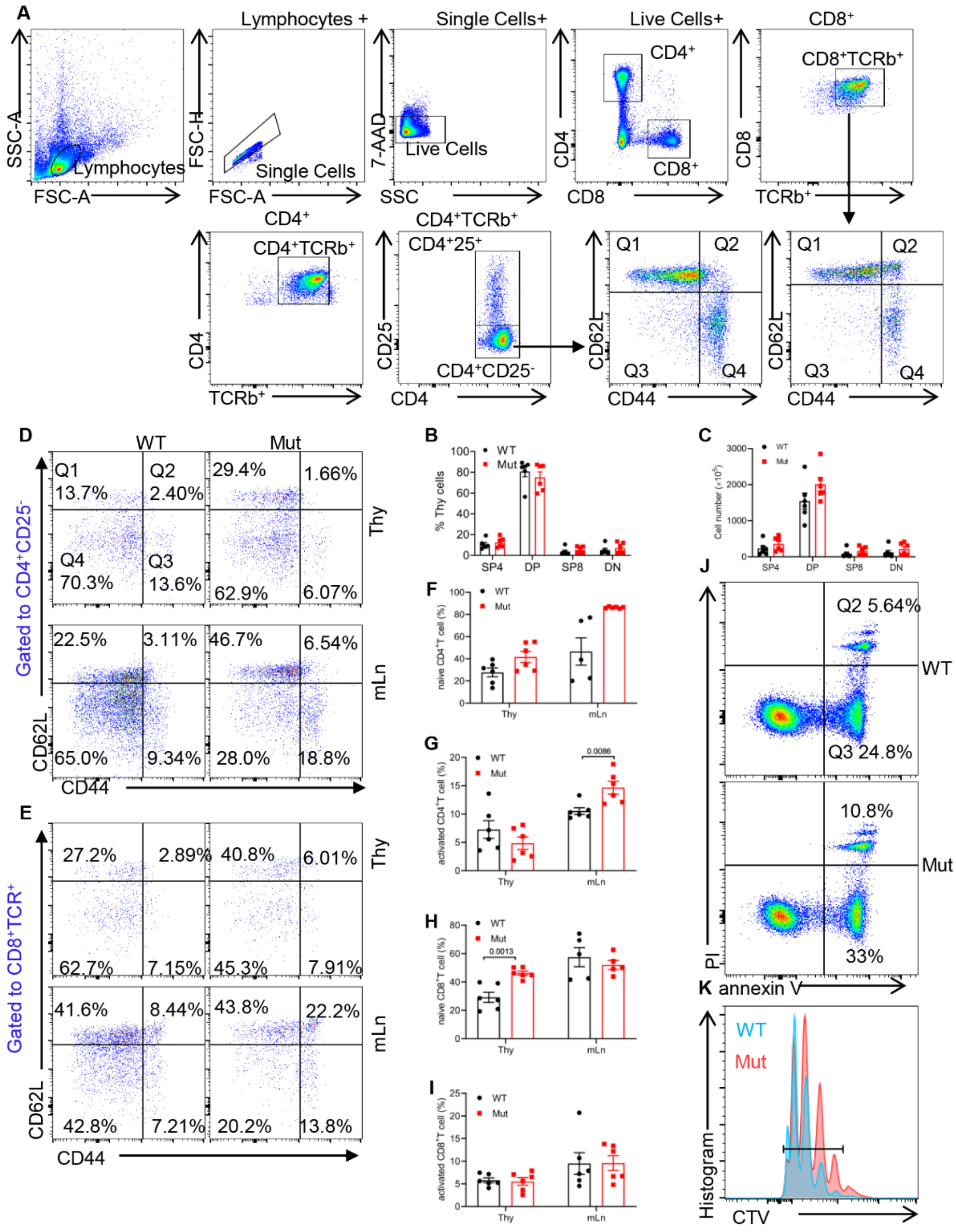

Figure S2. ***DOCK8*** **mutation impairs peripheral T cell homeostasis without affecting thymus T cell development.**

(A) The overall gating strategies for T cells of flow cytometry data. (**B-C)** Flow cytometry analysis of T cells in the thymus from WT and *DOCK8* mutant mice. Quantification of the percentages and cell numbers of T cells (SP4=CD4 single positive, SP8=CD8 single positive, DP=double positive, DN=double negative, n=6). (**D-I)** Flow cytometry analysis of naive T cells and activated T cells in the

thymus and mLn from WT and *DOCK8* mutant mice. Representative plots of flow cytometry (D-E). Quantification of the percentages of naive and activated T cells in the thymus and mLn (F-I, n=6). **(J)** Flow cytometry analysis of apoptotic $CD4^+$ T cells in the spleen from WT and *DOCK8* mutant mice. Representative plots of flow cytometry. **(K)** Proliferation of $CD4^+$ T cells in the spleen from WT and *DOCK8* mutant mice after stimulation with 10μg/ml plate-bound anti-CD3 plus anti-CD28 for 72 h at 37°C. Representative plots of flow cytometry. The data shown are the mean value ± SEM. *P < 0.05; **P < 0.01; ***P < 0.001.

Figure. S3.

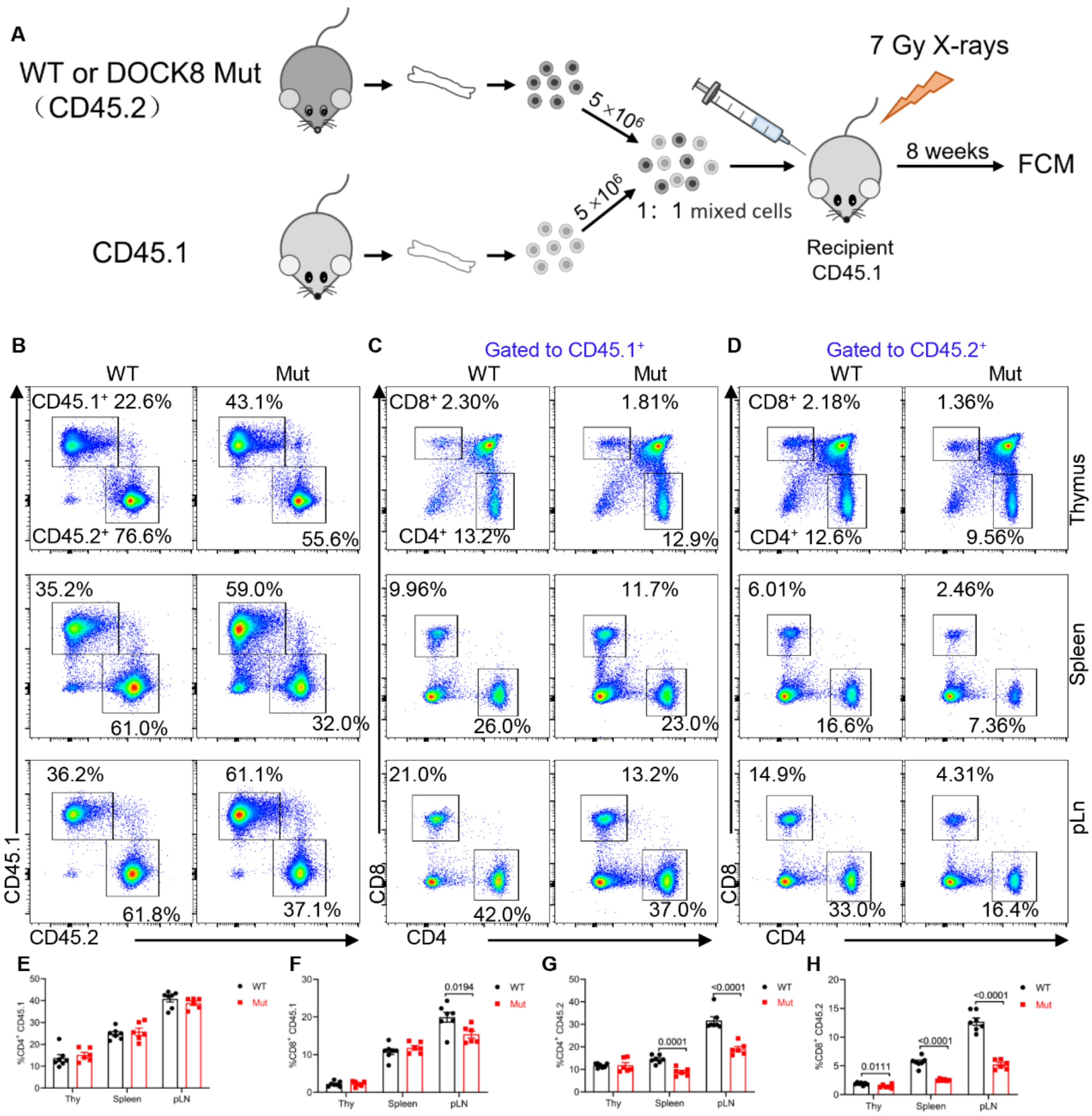

Figure S3. *DOCK8* mutation cripples peripheral T cell homeostasis in a cell-intrinsic manner.

(A) Schematic illustration of bone marrow chimeras. **(B-H)** Flow cytometry analysis of $CD4^+$ T cells and $CD8^+$ T cells in the thymus, spleen and mLn of mixed bone marrow chimeras eight weeks after transfer. Representative plots of flow cytometry (B-D). The percentages of $CD4^+$ T cells and $CD8^+$ T cells was quantified (E-H, n=7). The data shown are the mean value ± SEM. **P* < 0.05; ***P* < 0.01; ****P* < 0.001.

Table S1. The list of reagents and resources.

REAGENT or RESOURCE	SOURCE	IDENTIFIER
**Antibodies**		
FITC-anti-Annexin V	Biolegend	Cat#640906
PE-anti-CD278	Biolegend	Cat#107705
PE-anti-CTLA4	Biolegend	Cat#106306
PerCP-Cy5.5-anti-CD44	Biolegend	Cat#103032
APC-anti-CD25	Biolegend	Cat#102012
APC/Cy7-anti-TCR beta Chain	Biolegend	Cat#109220
PE/Cy7-anti-CD62L	Biolegend	Cat#104418
PE/Cy7-anti-CD279	Biolegend	Cat#109110
Pacific Blue-anti-CD4	Biolegend	Cat#100531
Brilliant Violet (BV)510-anti-CD8a	Biolegend	Cat#100751
7-AAD	BD Pharmingen™	Cat#559925
APC/Cy7-anti-CD45.1	Biolegend	Cat#110716
BV510-anti-CD45.2	Biolegend	Cat#109838
PE-Cy7-anti-ki67	eBioscience	Cat#25-5698-82
APC-anti-CD304	Biolegend	Cat#145206
AF488-anti-Foxp3	Thermo Fisher Scientific	Cat#53-5773-82
PE-anti-CD4	Biolegend	Cat#100408
PerCP/Cy5.5-anti-CD44	Biolegend	103032

APC-anti-IL-4	Biolegend	Cat#504106
PE/Cy7-anti-IFN-γ	Biolegend	Cat#505826
Brilliant Violet 421-anti-IL-17A	Biolegend	Cat#506925
Dock8	Proteintech	Cat#11622-1-AP
Anti-pAKT antibody	Cell Signaling Technology	Cat#9272S
Anti-AKT antibody	Cell Signaling Technology	Cat#9272S
Anti-pS6 antibody	Cell Signaling Technology	Cat#4856S
Anti-S6 antibody	Cell Signaling Technology	Cat#2217S
Anti-pmTOR antibody	Cell Signaling Technology	Cat#5536S
Anti-mTOR antibody	Cell Signaling Technology	Cat#2983S
Anti-HIF1a antibody	Active Motif	Cat#39665
Anti-PKM2 antibody	Cell Signaling Technology	Cat#4053T
Anti- β-actin antibody	Proteintech	Cat#60008-1-IG-10
Fc blocker	BioLegend	Cat#101319

anti-CD3 plus anti-CD28	Gibco	Cat#11131D
**Chemicals, peptides, and recombinant proteins**		
Red Cell Lysis Buffer	Tiagen	Cat#RT122-02
PMA	Sigma-Aldrich	Cat#P1585-1MG
GolgiStop	BD Biosciences	Cat#554724
Lonomycin	Cell Signaling Technology	Cat#9995S
DHE	Invitrogen	Cat#C10444
PK Mito Red	Genvivotech	Cat#PKMDR-1
RIPA buffer	Beyotime	Cat#P0013B
Cocktail	Servicebio	Cat#G2006
NaF	Servicebio	Cat# G2007-1
Na3VO3	Servicebio	Cat# G2007-1
Celltrace Violet	Thermo Fisher	Cat#C34557
Oligomyciine	Absin	Cat#abs4202430 4
Fluoro-carbonylcyanide phenylhydrazone	Sigma	Cat#C2920
Rotenone	Sigma	Cat#R8875
Antimycine	Absin	Cat#abs4201340
Glucose	Sigma	Cat#G8769
2-DG	Sigma	Cat#D8375

Trizol	Invitrogen	Cat#15596026
Attune performance tracking beads	Invitrogen	Cat#2029773
**Critical commercial assays**		
CD4$^+$ T cell Isolation Kit	Miltenyi	Cat#130-104-453
Fixation/Permeabilization Kit	eBioscience	Cat#00-5123, Cat#00-5223
IgE ELISA Kit	NeoBioscience	Cat#EMC117.96
RNA-seq Library Prep Kit	Vazyme	Cat#NR605-01
PrimeScript RT Reagent Kit	Takara	Cat#RR047A
SYBR Premix Ex TaqTM	Takara	Cat#RR420A
The HiScript® III 1st Strand cDNA Synthesis Kit	Vazyme	Cat# R211-02

Table S2. Primer sequences used in the study.

Primer	Forward	Reverse
**Real-time PCR primer sequences**		
GAPDH	GGTGAAGGTCGGTGTGAACG	CTCGCTCCTGGAAGATGGT G
IL1R1	CCCTGGCTTGTGTTACAGCA	AATGTGGAGCCGCTGTGG
SOX4	GACAGCGACAAGATTCCGTT C	GTTGCCCGACTTCACCTTC
RORC	ACAGCCACTGCATTCCCAGT TT	TCTCGGAAGGACTTGCAGA CAT
IL17F	CCCATGGGATTACAACATCA CTC	CACTGGGCCTCAGCGATC
